# CITY LIGHTS OR COUNTRYSIDE NIGHTS? RURALITY’S ROLE ON ANXIETY AND SLEEP IN MID- TO LATE-LIFE

**DOI:** 10.1093/geroni/igae098.1181

**Published:** 2024-12-31

**Authors:** Amy Costa, Puja Halder, Christina McCrae, Ashley Curtis

**Affiliations:** University of South Florida, Tampa, Florida, United States; University of Missouri, Columbia, Missouri, United States; University of South Florida, Tampa, Florida, United States; University of South Florida, Tampa, Florida, United States

## Abstract

Anxiety and sleep health associations are well studied but contributing factors (e.g., level of rurality) to health disparities in this relationship are understudied, particularly in aging populations. The present study examined the moderating impact of rurality on associations between anxiety and aspects of sleep health in middle-aged and older adults living in the United States. Cognitively healthy middle-aged and older adults (N=251; Mage=64.7±7.9 years) completed an online survey measuring residence zip code, Pre-Sleep Arousal Scale, Pittsburgh Sleep Quality Index, and the Hospital Anxiety and Depression scale. Multiple regression and simple slope analyses examined whether rurality (rural vs. non-rural; coded by Delineating Core Based Statistical Areas) moderated associations between anxiety and sleep, controlling for age, sex, education, income, depression, medication usage, and nutritional status (Mini-Nutritional Assessment). Results revealed rurality moderated the association between anxiety and cognitive pre-sleep arousal (R2 change=.014, p=.01) and sleep disturbances (R2 change=.01, p=.04). Higher anxiety was associated with higher cognitive pre-sleep arousal, with an ~2x larger association for rural (B=.64, p<.0001) than non-rural (B=.35, p=.0002) participants. Similarly, higher anxiety was associated with more sleep disturbances for rural (B=.05, p<.001), than non-rural participants. In mid-to-late life, those living in rural areas may be more vulnerable to the impact of higher anxiety on worse sleep compared to those living in non-rural areas. Findings point to the need for a greater understanding of socioeconomic determinants of health and more tailored treatments, with potential emphasis on mitigating barriers impacting rural access to clinical treatments for anxiety and sleep health.

